# A Host Tree and Its Specialist Insects: Black Locust (*Robinia pseudoacacia*) Availability Largely Determines the Future Range Dynamics of Its Specialist Insects in Europe

**DOI:** 10.3390/insects15100765

**Published:** 2024-10-02

**Authors:** Xueyou Zhang, Peixiao Nie, Xiaokang Hu, Jianmeng Feng

**Affiliations:** College of Agriculture and Biological Science, Dali University, Dali 671003, China; zhangxueyou0604@163.com (X.Z.);

**Keywords:** black locust, Europe, host plant availability, range dynamics, *Robinia*-specialist insects

## Abstract

**Simple Summary:**

Black locust (*Robinia pseudoacacia*) and its specialist insects have major effects on ecosystems in Europe, where the former is the only host of the latter. Here, we determined the relative roles of host plant availability and other predictors in shaping the specialist insects’ future range dynamics. Range expansions were detected in all target species. Climate predictors were expected to have the strongest effects on the range shifts of the host plant, while host plant availability had the strongest effect on the range shifts of the specialist insects. Therefore, range shifts in specialist insects tracked those of their host. Mitigating future climate change might be one of the key approaches for controlling invasions of *Robinia*-specialist insects in Europe, given that their range changes follow those of their specialist host plant, and future climate changes were mainly responsible for the range expansions of the host plant.

**Abstract:**

Black locust is the only host of *Robinia*-specialist insects in Europe. However, no study to date has examined future range shifts of specialist insects, and the relative effects of host plant availability and other factors on their range shifts. Here, we characterized the future range shifts in the host and its four specialist insects and the factors contributing to changes in their ranges. We detected substantial range expansions in all target species. Climate predictors and host plant availability were expected to have the strongest effects on the range shifts in the host and its specialist insects, respectively, suggesting that the specialist insects will track the ranges of their host. *Parectopa robiniella* showed the largest potential and expanding ranges and should be made a priority species for controlling invasions of *Robinia*-specialist insects in Europe. The expanding ranges of all specialist species were largely identified in the United Kingdom, Germany, and France, suggesting that these should be priority regions for mitigating their effects on ecosystems. Reducing future climate change is essential for preventing the spreading of specialist insects in Europe since specialist insects track their specialist host plants, and host range expansions are mainly driven by future climate changes.

## 1. Introduction

*Robinia pseudoacacia* L. (black locust), a tree species native to North America, has been introduced to most warm regions on all continents and is thought to have been cultivated as an ornamental tree in Europe since 1601 [1,2,3]. Owing to its fast growth, high-quality timber, resistance to air pollution, and large honey production, this tree species is considered the most economically important alien plant in Europe [1,4,5]. Nevertheless, despite its economic importance, its strong biological plasticity and propagation ability have had negative effects on ecosystem integrity, biodiversity, and plant communities in Europe [2,6,7,8,9,10,11]. Consequently, it has been listed as one of the “100 worst” alien species in Europe [10]. In light of its economic importance and invasiveness, its potential range and distribution patterns in Europe have received much attention, and these studies have provided important insights with implications for its control and cultivation [11,12,13,14].

Numerous factors likely affect the potential ranges of black locust in Europe. First, soil properties strongly affect the establishment and growth of black locust [15,16,17,18]. For example, Rédei et al. [19] have observed high establishment success of black locust at sites with well-aerated soil, with adequate moisture, appropriate texture, and rich in nutrients and humus. Therefore, soil factors might affect the potential ranges of black locust, although few studies have tested this hypothesis. Second, topographical factors may play important roles in shaping the potential ranges of black locust [4,20], and the roles of topographical factors have received much research attention. For example, Dyakov [21] found that topographical factors could affect the distribution of black locust in Central Western Bulgaria. Additionally, anthropogenic disturbance can promote the spread of black locust [20,22], and farmland abandonment, large-scale disturbances in urban and industrial areas, and human-induced landscape fragmentation have created large ecotones and linear woodland boundaries, which have facilitated the spread of black locust [23]. Black locust is thus a strong ecosystem transformer in disturbed landscapes [24] but a weak competitor in undisturbed sites [23]. Thus, anthropogenic disturbance factors might be one of the determinants of the potential range of this alien tree in Europe. However, few studies have examined this topic. Finally, several studies have suggested that climatic variables have strong effects on the potential ranges of black locust [4,12,13,14]. For example, Puchałka et al. [13] argued that future climate changes might induce eastward shifts in the potential ranges of this species and a substantial decline in Southern Europe. Undoubtedly, these studies have furthered our knowledge of the mechanisms underlying spatial variation in the potential ranges of this target tree in Europe. However, few studies have examined the relative effects of soil, climate, topography, and anthropogenic disturbance on the range dynamics of black locust in Europe under future scenarios.

More than 300 years after its introduction to Europe, six *Robinia*-specialist insects from North America, where black locust is native, were accidentally introduced to Europe: *Appendiseta robiniae* Gillette (Hemiptera: Drepanosiphidae), *Chrysaster ostensackenella* Fitch (Lepidoptera: Gracillariidae), and *Euura tibialis* Newman (Hymenoptera: Tenthredinidae), including *Obolodiplosis robiniae* Haldeman (Diptera: Cecidomyiidae), *Macrosaccus robiniella* Clemens (Lepidoptera Gracillariidae), and *Parectopa robiniella* Clemens (Lepidoptera: Gracillariidae) [12,13,16,25,26,27,28]. Since black locust is the main body of their food sources, their feeding could pose serious threats to the health status and primary production of the host tree [14,28,29,30]. Additionally, most of these specialist insects have rapidly expanded their ranges in Europe. For example, *P. robiniella* could spread at a speed of approximately 100 km per year [31]. Therefore, the *Robinia*-specialist insects in Europe have high invasiveness, and this could affect the black locust wood industry.

Owing to their high invasiveness, the spatial distributions of these species have received much research attention. Tytar et al. [14] projected the distribution of *P. robiniella* in Ukraine and found that the southwestern and western regions and Transcarpathia were priority regions for the invasion of this pest. Additionally, Zhao et al. [32] predicted the potential range of *O. robiniae* in Eurasia under future scenarios, and semi-humid and semi-arid regions comprised the main area of its potential range; they also detected a large-scale northward expansion. These efforts have enhanced our understanding of the hotspots of their invasions. However, range shifts of most specialist insects of black locust in Europe under future scenarios have not been studied to date using a unified scheme.

Given that these insects are specialists of black locust and no native *Robinia* plants occur in Europe [26,33], black locust is their only host in Europe. The spread of these specialist insects thus likely depends closely on black locust. Mally et al. [12] found that black locust had major effects in driving historical changes in the spatial dynamics of its specialist insects in Europe, and Medzihorský et al. [26] obtained similar conclusions at a global scale. We assumed that the potential ranges of its specialist insects in Europe might be primarily controlled by the availability of black locust. However, this assumption remains controversial. For example, Tytar et al. [14] found that climate predictors play a stronger role in determining the distributions of *P. robiniella* in Ukraine than the availability of black locust, whereas Mally et al. [12] found that black locust has had a stronger effect on the historical spatial dynamics of *Macrosaccus robiniella*, *P. robiniella*, and *O. robiniae* in Europe compared with climatic predictors. Therefore, additional studies of the relative influences of black locust availability and other predictors in determining the range dynamics of specialist insects are needed.

In addition to the effects of host plant availability and climatic predictors on the range dynamics of specialist insects, the roles of topographical factors and anthropogenic disturbance in shaping their potential ranges cannot be neglected. The former is closely linked to the formation of diverse habitats and generates barriers to species spread [34,35,36,37], while the latter can modify habitat suitability for most species [38,39,40], and both might affect the potential ranges of *Robinia*-specialist insects in Europe. Nonetheless, the relative effects of topography, host plant availability, climate, and anthropogenic disturbance on the future range shifts in *Robinia*-specialist insects of black locust in Europe have received little attention.

Here, we used soil, topographical, climatic, and anthropogenic disturbance predictors to predict the habitat suitability and range shifts of black locust in Europe in the future; we then projected the range of specialist insects of black locust in Europe and the relative roles of host plant availability and other factors. We hypothesized that the availability of black locust might have a stronger effect on the range dynamics of the specialist insects relative to other factors, and the main body of the potential ranges of these specialist insects might fall within those of black locust. Our findings provide novel insights that could help policymakers prevent the spread of specialist insects of black locust in Europe, as well as improve the cultivation and control of black locust in this region.

## 2. Materials and Methods

### 2.1. Retrieving Occurrence Records

**Figure 1:**
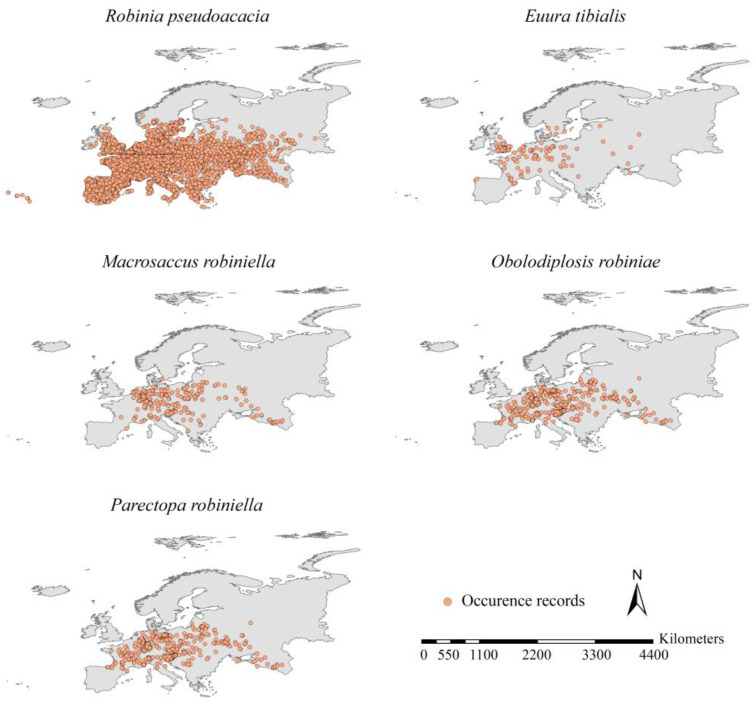
Occurrence records of the five target species. After spatial thinning, a total of 22,093 occurrences were obtained, with 20,790, 118, 279, 353, and 553 records of *Robinia pseudoacacia*, *Euura tibialis*, *Macrosaccus robiniella*, *Obolodiplosis robiniae,* and *Parectopa robiniella*, respectively.

We retrieved five black locust-specialist species from the literature [12,26,33,41]: *C. ostensackenella*, *O. robiniae*, *P. robiniella*, *M. robiniella*, *Euura tibialis*, and *Appendiseta robiniae*. Following a method suggested by Mally et al. [12], we searched the occurrence records of black locust and its specialist insects in literature and online datasets (including GBIF (https://www.gbif.org (accessed on 15 March 2024)), EPPO (https://gd.eppo.int (accessed on 21 March 2024)) and CABI (https://www.cabi.org/ISC (accessed on 23 March 2024))). Additionally, we also incorporated checklists of occurrence records of the specialist insects compiled by Mally et al. [12] into our occurrence dataset. We obtained a total of 324,842 records of the locust and its specialist insects in Europe. We then compiled an occurrence dataset for each species. Due to the scarcity of occurrence records in Europe (fewer than 30 records), *A. robiniae* and *C. ostensackenella* were omitted from our investigation. Following a study by Zhang et al. [42], we removed any occurrence records with geographic coordinate uncertainty greater than 5 km. Additionally, we retained only one record in each grid cell of 5 × 5 km using the spatial thinning method proposed by Zhang et al. [42]. We obtained a final occurrence dataset for each species with 22,093 records, including 20,790, 118, 279, 353, and 553 records for black locust, *E. tibialis*, *M. robiniella*, *O. robiniae*, and *P. robiniella*, respectively (Figure 1 and Supplementary Material S1).

### 2.2. Preparing Predictors

We prepared four groups of 46 predictors to predict potential ranges and habitat suitability for black locust. These included climate (19), soil properties (16), land use (8), and topography (3) predictors (Supplementary Material S2). The climatic predictors under current scenarios comprised 19 climatic variables from 1990 to 2020 at the European scale. We downloaded monthly averaged precipitation and temperature variables at a spatial resolution of 2.5 arc-minutes from the Climate Research Division (CRU, https://crudata.uea.ac.uk/, accessed on 3 October 2023). Next, we used the ‘Biovarcs’ R package [43] to calculate 19 climatic predictors under current scenarios, which matched the 19 climatic variables in Worldclim [43] (Supplementary Material S2). The 19 future climate variables under future scenarios (in 2100) were obtained from Worldclim (www.worldclim.org, accessed on 3 October 2023). They were derived through two robust and complementary global circulation models: MPI-ESM-HR (MPI) and FIO-ESM-2 (FIO) [44]. We examined two scenarios in the future, Shared Socio-Economic Pathways 585 and 126, representing the pessimistic and optimistic climate change scenarios, respectively. We retrieved a total of five datasets of climate predictors: current scenarios under current conditions, SSP126 scenario derived by FIO (FIO126), SSP585 scenario derived by FIO (FIO585), SSP126 scenario derived by MPI (MPI126), and SSP585 scenario derived by MPI (MPI585). The 16 soil property variables with a spatial resolution of 0.5 arc-minutes were retrieved from the Harmonized World Soil Database (https://previous.iiasa.ac.at/web/home/research/researchPrograms/water/HWSD.html, accessed on 3 October 2023) (Supplementary Material S2); we then resampled the 16 soil property variables to a 2.5 arc-minute spatial resolution. We used land use predictors to represent anthropogenic disturbance, and they comprised eight land use factors (Supplementary Material S2). Initially, we downloaded them at a spatial resolution of 0.25 arc-degrees from the Land-Use Harmonization online dataset (LUH2, https://luh.umd.edu/, accessed on 3 October 2023). They were then resampled at a spatial resolution of 2.5 arc-minutes. Additionally, they had three datasets: current land use variables, those under the SSP126 scenario in 2100, and those under the SSP585 scenario in 2100. Data on topographical factors, including elevation, aspect, and slope, were obtained from the Worldclim database (www.worldclim.org, accessed on 3 October 2023). They were derived from a digital elevation model at the European scale with a 0.5 arc-minute spatial resolution and then were resampled to 2.5 arc-minutes. Predictors of soil properties and topography remained constant under all scenarios.

The predictors for the ranges of the four specialist herbivores included four categories of 31 predictors: habitat suitability (1), topography (3), land use (8), and climate (19) (Supplementary Material S2). Topography, land use, and climate variables were the same as those used for projecting the potential ranges of black locust. Our black locust habitat suitability values were calibrated to represent the host plant availability of the specialist insects and were obtained through species distribution models (SDMs) built to predict habitat suitability and the range of black locust under all scenarios. Habitat suitability of black locust included five maps, including those under the current, FIO126, FIO585, MPI126, and MPI585 scenarios.

### 2.3. Predictor Selection

We used the following methods to reduce multi-collinearity among predictors. First, we built preliminary SDMs into which all predictors were input for each species. Next, we determined the importance value (IV) of each predictor using the jackknife technique (Supplementary Material S2). We also applied the method of Zhang et al. [42] to each species individually and conducted Pearson correlation analyses to examine the collinearity among predictors using a correlation coefficient threshold of |0.7| (Supplementary Material S3). The variable with the lower IV was removed if strong collinearity was identified between any two variables (Supplementary Materials S3). Finally, we inputted the retained variables into the formal SDMs to calculate the potential range of each species.

### 2.4. SDM Development

We built five SDMs to calculate the ranges of the five species. We projected the ranges of the species using the R package ‘Biomod2’ [45], a platform for building ensemble SDMs, in which we used 10 algorithms (Supplementary Material S4). As required by presence-only SDMs, a five-step selection process was used to obtain pseudo-absences (PAs) [45,46]: we randomly selected 1000 PAs across Europe when the number of occurrences of a species was less than 1000, or we randomly selected PAs equal to the number of occurrences. Using the occurrences, we assessed the robustness of the SDMs through five-fold cross-validation, with seven folds randomly selected for developing SDMs, and the remaining three folds for assessing the reliability of the SDMs [45,47]. To ensure high model performance, we followed a method suggested by Nie and Feng [48], in which SDMs derived by algorithms with true skill statistic values lower than 0.6 and the values of area under the curve smaller than 0.8 were not incorporated into the ensemble platform (Supplementary Material S5). The ensemble SDM platform initially projected the habitat suitability maps of each species, and the maximum-sensitivity–specificity-sum threshold (MSS threshold) [49] was applied to maps of the black locust’s habitat suitability for determining the potential range of the black locust. Under each scenario, we overlapped the potential ranges of black locust with the ranges of each specialist herbivore. We also estimated the percentages of the above-mentioned overlapped ranges in the ranges of the specialist insects for each species and scenario:

$$POR=\frac{S}{RH},$$

where *POR* is the percentage of the overlapped range in the range of the specialist insect, and *S* and *RH* indicate the overlapped range between the black locust and its specialist herbivore and the range of the specialist insect, respectively.

### 2.5. Range Dynamics

We built range dynamic models to examine range shifts in the black locust and its specialist insects. We estimated the range expansion ratio (*RER*) and index of range similarity (*IRS*) to examine range dynamics for each species.

The *RER* was built to project the shifts in range sizes between current scenarios and future ones:

$$RER=\frac{RF}{RC},$$

in which *RC* and *RF* were the ranges under current scenarios and future ones, respectively.

We created *IRS* to indicate the shifts in the range centroids:

$$IRS=\frac{2RS}{RC+RF},$$

in which RS is the range shared by *RF* and *RC*. If *IRS* > 0.5, *RF* and *RC* had similar range centroids.

For each species, we also predicted the range that it could possibly exploit only under future scenarios (i.e., expanding range) by overlapping current ranges with those in the future.

### 2.6. Statistical Analysis

For each target species, Pearson correlation analysis was conducted to retrieve correlation coefficients between each pair of variables. Next, we adopted a coefficient threshold of |0.7| to determine collinearity among all the predictors [39]. We applied paired-sample *t*-tests to compare the range dynamics of the target species under current scenarios with those in the future.

## 3. Results

### 3.1. Model Reliability

Owing to our ensemble SDM platform, our SDMs showed high performance with high values of the area under the curve and true skill statistics. Specifically, the true skill statistic values for the five species varied from 0.79 to 0.90, with an average of 0.85 ± 0.05 (Supplementary Material S6). Values of the area under the curve for the five species varied from 0.96 to 0.98, with an average of 0.97 ± 0.01 (Supplementary Material S6). All these observations suggest that our SDMs were effective in clarifying range shifts in our target species under future scenarios.

### 3.2. Predictors Affecting Range Dynamics

**Figure 2:**
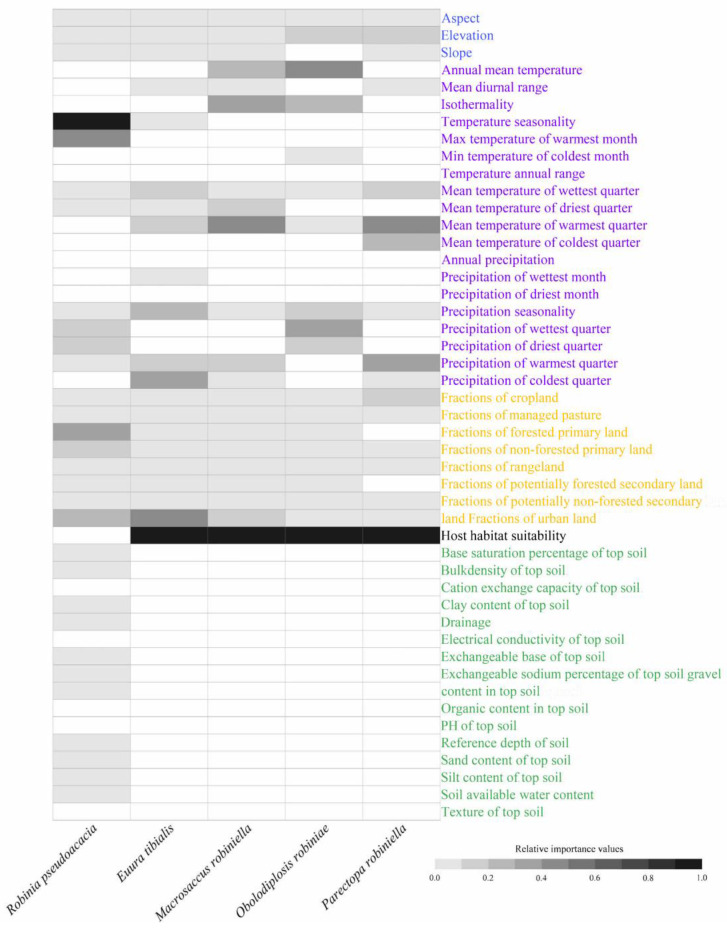
Variable importance in the formal models. Factors on topography, climate, land use, host habitat suitability, and soil were indicated in blue, purple, yellow, black, and green fonts, respectively. We standardized the importance scores through the max–min method. Our grayscale indicated the relative scores of variable importance, and the blanks meant that the variables were not inputted into the formal models.

The most important predictors responsible for the range dynamics of black locust were temperature seasonality (IV of 0.419), followed by maximum temperature in the warmest month (0.092) and primary forested land (0.040) (Figure 2, Supplementary Material S7). Additionally, topography and soil property-related predictors were not among the 10 most important predictors (Figure 2 and Supplementary Material S7). The most important predictor of the range dynamics of the four specialist insects was the habitat suitability of their host (black locust) (Figure 2, Supplementary Material S7). Moreover, the IVs of the habitat suitability of their host were far higher than those of the other predictors, which were indicated by comparisons between the highest and second highest IVs for each species: 0.739 vs. 0.068, 0.437 vs. 0.113, 0.613 vs. 0.109, and 0.413 vs. 0.182 for the potential ranges of *E. ibialis*, *M. robiniella*, *O. robiniae*, and *P. robiniella*, respectively (Supplementary Material S7). The most consistently important predictor of the ranges of all specialist insects was habitat suitability. The second most important predictors varied among species and included the proportion of urban land, mean temperature of the warmest quarter, mean annual temperature, and mean temperature of the warmest quarter for the potential ranges of *E. tibialis*, *M. robiniella*, *O. robiniae*, and *P. robiniella*, respectively.

### 3.3. Habitat Suitability of Black Locust and Its Specialist Insects

**Figure 3:**
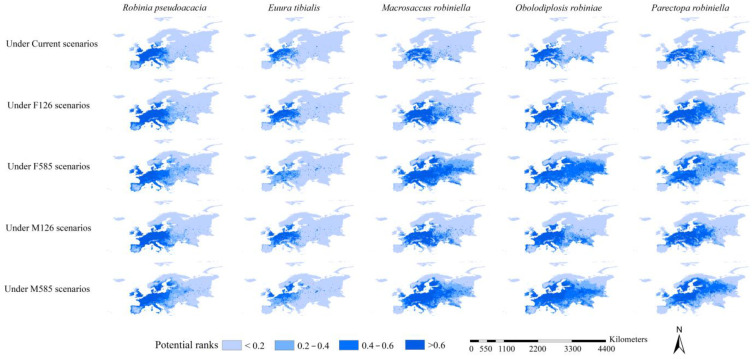
Habitat suitability of *Robinia pseudoacacia* and its specialist insects. Habitat suitability > 0.6, 0.4–0.6, 0.2–0.4, and <0.2 were defined as high, medium, low, and extremely low habitat suitability, respectively. F126, FIO126; F585, FIO585; M126, MPI126; M585, MPI585. High habitat suitability of all species was mostly detected in the United Kingdom, France, and Germany.

Under current and FIO126, MPI126, and FIO585 scenarios, high habitat suitability (>0.6, same as below) of black locust was mostly projected in France, the United Kingdom, and Germany; under MPI585, high habitat suitability was generally observed in France, the United Kingdom, Germany, and Sweden (Figure 3). Under all scenarios, low habitat suitability (<0.4, same as below) was mainly detected in West Russia, East Europe (except Poland), North Europe (except Sweden), and South Europe (Figure 3). The distribution of high habitat suitability of *E. tibialis* under all scenarios was scattered throughout Europe and primarily detected in the United Kingdom, Germany, France, and Poland (Figure 3). Under current, FIO126, and MPI126 scenarios, high habitat suitability for *M. robiniella* was largely predicted in Germany, France, Austria, and Poland (Figure 3). Under the FIO585 and MPI585 scenarios, high habitat suitability was primarily predicted in the United Kingdom, Germany, France, Poland, Austria, Switzerland, Sweden, and West Russia (Figure 3). Under all scenarios, the distribution of high habitat suitability for *O. robiniae* was similar to that for *M. robiniella* (Figure 3). Under current conditions and the FIO126 and MPI126 scenarios, areas with high habitat suitability for P. robiniella exhibited a similar pattern, and they were mainly detected in France, Germany, Poland, the Czech Republic, Switzerland, Austria, Croatia, and Slovakia (Figure 3). Under the FIO585 and MPI585 scenarios, high habitat suitability was mainly identified in Sweden, Finland, Poland, Lithuania, France, Germany, the Czech Republic, Switzerland, Austria, and Slovakia (Figure 3). The distribution of areas with low habitat suitability for the specialist insects was similar to that for black locust (Figure 3).

### 3.4. Potential Ranges of Black Locust and Its Specialist Insects

The MSS threshold used to determine the ranges depended on species and scenarios (Supplementary Material S8). For instance, in the FIO585 scenario, the MSS threshold for *M. robiniella* and *P. robiniella* was 0.81 and 0.60, respectively (Supplementary Material S8). Additionally, the MSS threshold for *O. robiniae* under the FIO585 and MPI126 scenarios was 0.69 and 0.84, respectively (Supplementary Material S8).

**Figure 4:**
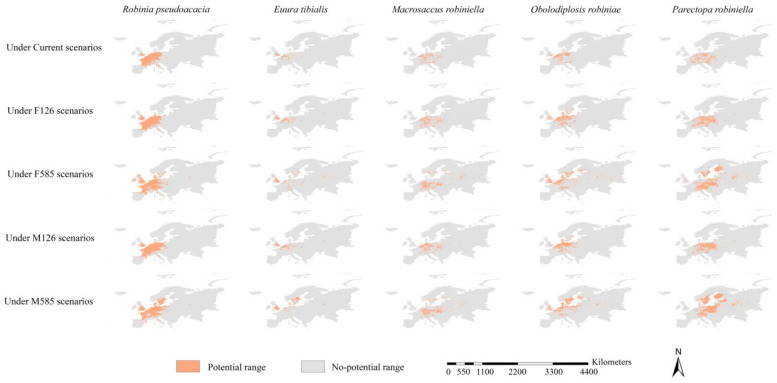
Potential ranges of *Robinia pseudoacacia* and its specialist insects. F126, FIO126; F585, FIO585; M126, MPI126; M585, MPI585. Under all scenarios, the potential ranges of *Robinia pseudoacacia* were largely projected to occur in the United Kingdom, France, and Germany. Those of *Euura tibialis*, *Macrosaccus robiniella,* and *Obolodiplosis robiniae* were mostly projected in the potential ranges of the black locust and in scattered patterns.

**Figure 5:**
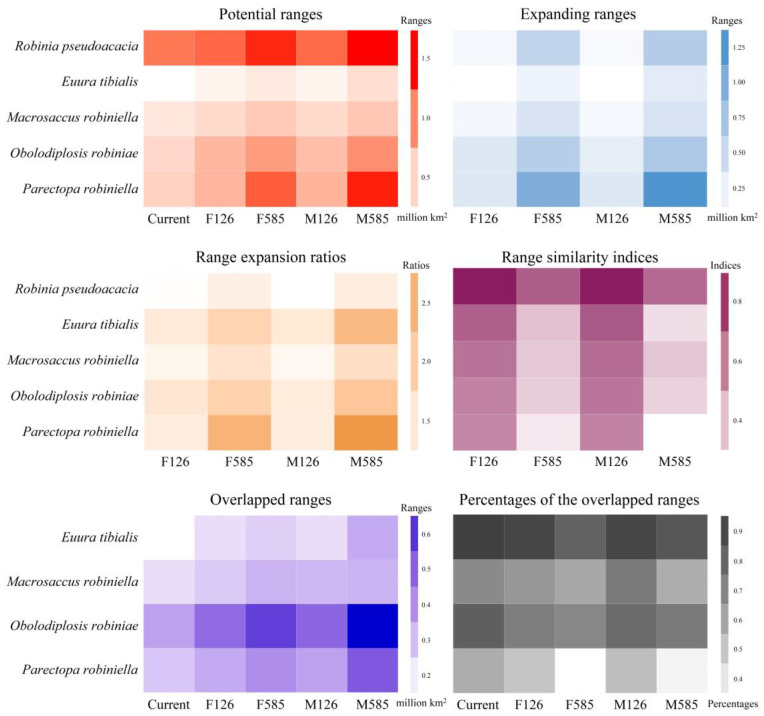
Areas of the ranges, expanding ranges, range expansion ratios, range similarity indices, areas of the overlapped ranges, and the overlapped range percentages. F126, FIO126; F585, FIO585; M126, MPI126; M585, MPI585. They were in red, blue, yellow, crimson, purple, and grey, respectively. *Robinia pseudoacacia* was projected to show the largest ranges. Among four specialist insects, *Parectopa robiniella* under all scenarios covered the largest ranges and expanding ranges. The ranges, future expanding ranges, and overlapped ranges were larger than current ones, and those under 585 scenarios were larger than those under 126 scenarios. The indices of range similarity under 126 scenarios were higher than 0.50, while those under 585 scenarios were smaller than 0.50.

Under the current scenarios, the ranges of the black locust were mainly projected in France and Germany; its potential range, which covered 1.07 million km^2^, was also scattered in various areas in Poland, Italy, Switzerland, and the United Kingdom (Figure 4 and Figure 5). Under the FIO126 and MPI126 scenarios, the ranges of black locust were similar to those under current scenarios, covering 1.17 and 1.15 million km^2^, respectively (Figure 4 and Figure 5). Relative to the current ranges, the future ranges of the black locust under the FIO585 and MPI585 scenarios extended further west in the United Kingdom, further east in East Europe, and further north in North Europe, covering 1.44 and 1.50 million km^2^, respectively (Figure 4 and Figure 5). The potential ranges of *E. tibialis*, *M. robiniella*, and *O. robiniae* under the five scenarios were all roughly within the potential ranges of the black locust under the corresponding scenarios, with the exception of a few scattered areas (Figure 4). The current range of *P. robiniella* was scattered in various areas and extended from France to West Russia and from Italy to Lithuania, covering 0.49 million km^2^ (Figure 4 and Figure 5). The potential ranges of *P. robiniella* under the FIO126 and MPI126 scenarios were roughly similar in size to those under the current scenario (except that the former was more concentrated), covering 0.68 and 0.69 million km^2^, respectively (Figure 4 and Figure 5). Relative to the current range, the future ranges of *P. robiniella* under the FIO585 and MPI585 scenarios extended further north in Sweden and Finland and east in Poland, Belarus, and West Russia, covering 1.22 and 1.46 million km^2^, respectively (Figure 4 and Figure 5). In sum, relative to the specialist insects, the ranges of black locust were projected to cover larger areas under all scenarios. Additionally, the potential range of *P. robiniella* was the largest among all four pests under all scenarios, followed by *O. robiniae* and M. robiniella.

Our paired sample *t*-tests indicated that the current ranges for all species were significantly smaller than those in the future (all *p* < 0.05), and the ranges under the FIO585 and MPI585 scenarios were significantly larger than the ranges under the FIO126 and MPI126 scenarios (all *p* < 0.05).

### 3.5. Range Dynamics of the Black Locust and Its Specialist Insects

The range expansion ratios of all species, which compare the range sizes between current and future scenarios, were higher than 1.00, suggesting that the ranges of our five target species are projected to expand in the future (Figure 5). Under most scenarios, the highest range expansion ratios were observed for *P. robiniella* (Figure 5). The indices of range similarity of species, which measured shifts in their range positions, varied among species and scenarios (Figure 5). The indices of range similarity of black locust under all scenarios were higher than 0.50, and those under the 126 scenarios were higher than those under the 585 scenarios (Figure 5). For all four specialist insects, the indices of range similarity under the 126 scenarios were higher than 0.50, whereas those under the 585 scenarios were smaller than 0.50 (Figure 5).

**Figure 6:**
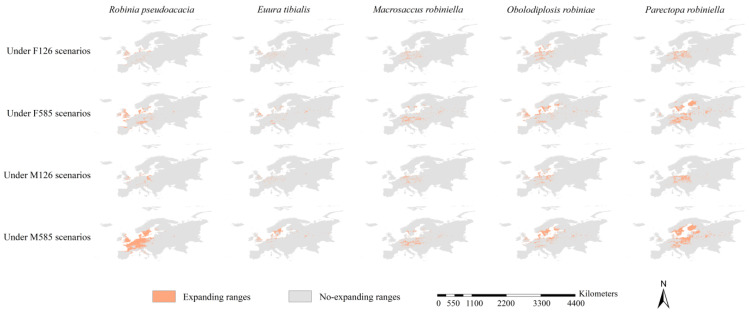
Expanding ranges of the host tree and its specialist insects. The expanding ranges of the black locust under FIO126 and MPI126 scenarios were in scattered patterns, extending in longitude from the United Kingdom to Lithuania and in latitude from Spain to Sweden. Its expanding ranges under FIO585 and MPI585 scenarios were mainly detected in Denmark, Sweden, Finland, Poland, and the United Kingdom. The expanding ranges of the four specialist insects varied with scenarios and species. The expanding ranges of *Euura tibialis*, *Macrosaccus robiniella,* and *Obolodiplosis robiniae* were in scattered patterns, and their main bodies were roughly in line with those of the black locust.

The expanding ranges of the black locust under the FIO126 and MPI126 scenarios were scattered in various areas extended from the United Kingdom to Lithuania and from Spain to Sweden, covering 0.17 and 0.16 million km^2^, respectively (Figure 5 and Figure 6). Its expanding ranges under the FIO585 and MPI585 scenarios were mainly detected in Denmark, Sweden, Finland, the United Kingdom, and Poland, covering 0.55 and 0.64 million km^2^, respectively (Figure 5 and Figure 6). The expanding ranges of the four specialist insects varied among scenarios and species. The expanding ranges of *E. tibialis*, *M. robiniella*, and *O. robiniae* were scattered in various areas, and they were mainly located within the potential ranges of black locust under their corresponding scenarios (Figure 6). Under the FIO126 and MPI126 scenarios, the expanding ranges of *P. robiniella* were primarily detected in Germany, Poland, France, Slovakia, and Austria (Figure 6). The expanding ranges under the FIO585 and MPI585 scenarios were mainly detected in Finland, Sweden, Poland, Lithuania, Latvia, and Germany. Generally, the ranges of our target species expanded northward and eastward. The paired sample *t*-tests revealed that the expanding ranges of all five species were larger under the 585 scenarios than under the 126 scenarios (all *p* < 0.05). Under all future scenarios, *P. robiniella* was predicted to have the largest expanding ranges among the four specialist insects (0.35, 0.99, 0.34, and 1.27 million km^2^ under the FIO126, FIO585, MPI126, and MPI585 scenarios, respectively), and the smallest expanding range was observed for *E. tibialis* (0.10, 0.22, 0.10, and 0.31 million km^2^ under the FIO126, FIO585, MPI126, and MPI585 scenarios, respectively) (Figure 5).

### 3.6. Range Overlap between Black Locust and Its Specialist Insects

**Figure 7:**
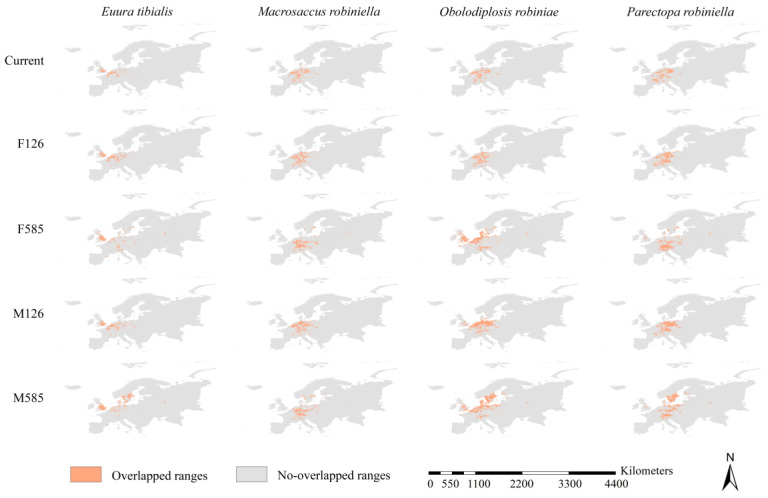
Overlapped between the specialist insects and their host tree. F126, FIO126; F585, FIO585; M126, MPI126; M585, MPI585. The overlapped range of *Euura tibialis*, *Macrosaccus robiniella,* and *Obolodiplosis robiniae* with their host tree were mainly detected in the ranges of the black locust. The overlapped ranges under the scenarios of FIO126 and FIO585 between *Parectopa robiniella* and its host tree were primarily detected in France and Germany. Under the scenarios of MPI126, the overlapped ranges were largely identified in Germany and Poland, and under the scenarios of MPI585, they were largely identified in Germany, Sweden, and Poland.

The sizes of the overlapped ranges between the black locust and its specialist insects varied among species and scenarios (Figure 5 and Figure 7). For example, the sizes of the overlapped ranges varied from 0.23 to 0.49 million km^2^ under the MPI126 scenario (Figure 5 and Figure 7). The paired sample *t*-test revealed that all current overlapped ranges were smaller than future ones (all *p* < 0.05), and the overlapped ranges were larger under the 585 scenarios than under the 126 scenarios (all *p* < 0.05). Additionally, under all scenarios, the smallest overlapped ranges were observed for *E. tibialis*, and the largest overlapped ranges were observed for *O. robiniae* (Figure 5 and Figure 7). The percentages of range overlap between the potential ranges of black locust and the potential ranges of *E. tibialis*, *M. robiniella*, and *O. robiniae* were higher than 57%, with an average of 75.8 ± 11% (Figure 5 and Figure 7). The range overlap percentages between black locust and *P. robiniella* were smaller compared with the range overlap percentages between black locust and the other three specialist insects, which ranged from 33% to 57% with an average of 46 ± 11%. Consistent with the high range overlap percentages observed, the overlapped ranges of insects were mainly projected within the potential ranges of the host (Figure 7). However, a substantial fraction of the overlapped ranges between the black locust and *P. robiniella* was primarily identified beyond the potential ranges of the black locust (Figure 7). Under current, FIO126, and FIO585 scenarios, the overlapped ranges between black locust and *P. robiniella* were largely predicted in France and Germany, and covered 0.28, 0.34, and 0.40 million km^2^, respectively (Figure 5 and Figure 7). Additionally, under the MPI126 scenario, the overlapped ranges were largely identified in Germany and Poland, and covered 0.36 million km^2^ (Figure 5 and Figure 7); under the MPI585 scenario, they were largely identified in Germany, Poland, and Sweden, and covered 0.52 million km^2^ (Figure 5 and Figure 7).

## 4. Discussion

We projected the range dynamics of the black locust and its four specialist insects in Europe, the range overlap between them, and the factors affecting changes in their potential ranges. Our study showed that host plant availability had stronger effects on determining the range dynamics of its specialist insects compared with other predictors, and major parts of the potential ranges of most specialist insects were within their host’s range. In other words, the specialist insects tracked their host plant, which was mainly because their host plant provided essential food resources and habitats [12,40]. Therefore, preventing the spread of black locust is essential for controlling the invasions of these specialist insects in Europe. Black locust has both beneficial and negative effects in Europe [1,3,33,50]; consequently, our findings have double-edged implications. For planters of black locust, our findings indicate that black locust will be continuously affected by its specialist insects given that the specialist insects are projected to track their host tree under most scenarios. Therefore, the effects of specialist insects on their host tree require increased attention. However, for policymakers who aim to design policies to limit the invasion of black locust, our findings suggest that specialist insects will mitigate some of the effects of black locust. Our study thus provides novel information that could aid the management of black locust and its specialist insects in Europe.

Tytar et al. [14] found that climate predictors play a stronger role in determining the distribution of *P. robiniella* in Ukraine relative to the availability of black locust. This could be explained by our finding that this insect could spread eastward as far as Ukraine, and its host occurs in areas further east than *P. robiniella*. This suggests that although host plant availability could facilitate its further spread to the east, environmental conditions, especially climatic conditions in Ukraine, impede its ability to spread. This might explain, at least in part, the stronger roles of climatic predictors compared with host plant availability. However, this was not the case in our European-scale study. Therefore, the relative effects of climatic predictors and black locust habitat suitability are affected by the spatial scale. Additional investigation of this hypothesis is needed.

A variety of variables have been used to project the range dynamics of most invasive herbivore insects, such as climate [50,51], topography [52,53], and land use variables [54,55], as well as host plant availability [56,57]. The black locust and its specialist insects provide a valuable opportunity to study the effects of host plant availability on the range shifts in herbivore insects relative to other predictors. We found that host plant availability plays a stronger role in determining range shifts in its specialist herbivore insects than other variables. This finding, to a certain extent, is consistent with the results of Mally et al. [12] showing that black locust occurrence plays a more important role in determining historical spatial shifts in *P. robiniella* in Europe than other predictors. Another study by Acosta and Mooney [58] on specialist species from another taxonomic group revealed similar patterns; specifically, they found that the availability of *Asclepias* spp. (the host plant) largely determined the distribution of *Danaus plexippus* (a specialist herbivore insect). Therefore, the distribution of specialist herbivore insects might be largely determined by host plant availability. This observation may also apply to non-specialist herbivore insects and their host plants. For example, Zhou et al. [57] found that the availability of the host plant *Prunus serotine* has a stronger effect on the distribution of the non-specialist herbivore insect *Drosophila suzukii* in Europe compared with other variables. Additionally, the results of Liu et al. [59] suggested that the potential ranges of *Spodoptera frugiperda*, a widely familiar polyphagous herbivore insect, were mostly shaped by host plant availability. Therefore, host plant availability may also play a key role in determining the ranges of non-specialist insects; generally, the proliferation of host plants might be largely responsible for the invasion of herbivorous insects [60]. Further studies should be conducted to confirm this argument.

The relative effects of land use and climate predictors on the species ranges vary with spatial scale; climate predictors tend to have stronger effects at large scales, whereas land use predictors tend to have stronger effects at small scales [61]. However, we found that their relative effects varied substantially among the five species, and our study was conducted at a single spatial scale. Therefore, their relative effects on the species ranges may depend on spatial scale and vary among species.

The factors affecting the future range dynamics of our target species might be closely associated with their biological traits. The physiological activities, phenological traits, growth processes, and reproduction of black locust are most strongly controlled by climatic conditions [62,63,64,65]. Therefore, relative to other factors, climatic predictors played stronger roles in determining its future range dynamics. Specialist insects are primary consumers in forest ecosystems, and their survival strongly depends on the food resources offered by *Robinia* trees. In our study, the black locust is the only host tree that could provide food resources for *Robinia*-specialist insects in Europe [26,30]. Thus, the availability of black locust had a stronger effect on the future range dynamics of the specialist insects relative to other factors.

Mally et al. [12] used five predictors to analyze the historical spread of the three specialist insects of black locust in Europe through reduced Cox proportional hazard models and found that the occurrence of black locust has had a strong effect on the historical spread of the three specialist insects in Europe. More recently, Medzihorský et al. [26] found that the richness of *Robinia*-specialist insects in North America could be jointly explained by the effects of road density, climate, and other factors on the occurrence of black locust using a negative binomial regression model. We used ensemble SDMs and 31 candidate predictors to determine range shifts of the four *Robinia*-specialist insects under current–future scenarios in Europe and the range overlap between the insects and their black locust hosts. We found that host plant availability had strong effects on their range dynamics. In contrast to previous studies, our findings provide important information on priority regions that will aid strategy makers to prevent the invasion of *Robinia*-specialist insects in Europe in the future, and assess their invasion risks under current–future scenarios. Therefore, this study provides essential information that will aid efforts to combat the invasion of *Robinia*-specialist insects in Europe.

Under all scenarios, *E. tibialis* was projected to have the smallest overlapped ranges with its specialist host, suggesting that this pest has weaker effects on its host relative to other specialist insects. However, *O. robiniae* had the largest overlapped range, indicating that this pest has a stronger effect on its specialist host relative to others, suggesting that *O. robiniae* merits more attention compared with other *Robinia*-specialist insects in Europe.

Under all scenarios, major parts of the potential ranges of *E. tibialis*, *M. robiniella*, and *O. robiniae* fell within the potential ranges of their specialist host tree. However, under most scenarios, more than 50% of the potential ranges of *P. robiniella* were not within the potential ranges of its host tree. These differences might be due to the fact that the role of host plant availability in the potential ranges of *P. robiniella* was more strongly shadowed by the effects of predictors other than host habitat suitability, especially climate variables, compared with those of the other three specialist insects. This is consistent with our observations that differences between the values of the strongest predictor (habitat suitability of the black locust) and the second-strongest predictor (mean temperature of the warmest quarter) in the models for *P. robiniella* were smaller than those for other specialist insects. Therefore, the extent to which specialist insects track the ranges of their host might vary among species.

Under the current–future scenarios, the black locust was projected to expand into some parts of the United Kingdom, Sweden, and France, et al., and climate change had the strongest effects on their range expansion. For black locust planters, these observations suggest that future climate change could promote efforts to expand the cultivation of this tree. Additionally, these regions might be suitable for black locust cultivation in the future, which would be beneficial to planters in these regions. However, for policymakers aiming to control the spread of black locust, reducing climate change in the future will be essential for mitigating its effect on European ecosystems, and increased efforts to control the spread of these invasive species in these regions will be needed in the future to minimize their effects on ecosystems in Europe. Range expansions of the four specialist insects were also projected under current–future scenarios, and these were mainly induced by range expansions of black locust. Therefore, reducing future climate change will be essential for combatting the invasion of specialist insects in Europe.

The main areas of the overlapped ranges between black locust and most of its specialist insects were detected in the United Kingdom, Sweden, France, et al. Therefore, these regions will require special attention to ensure that the effects of these specialist insects on black locust are minimized if maximizing the economic utility of this tree is made a priority. The results of this study demonstrated that *E. tibialis* had the smallest ranges and expanding ranges in the future among the four *Robinia* specialist insects in Europe. Therefore, this specialist insect may have the smallest invasion risk under all scenarios compared with the other species, indicating that it should not be made a priority species in efforts to control invasions of *Robinia*-specialist insects in Europe. However, *P. robiniella* likely poses the largest invasion risk; it should thus be made a priority species for control, and its invasion in Europe requires much more attention compared with that of other species in the future.

Finally, several limitations of our study require consideration. First, given that our range projections are theoretical, our projections might not be fully supported by the actual ranges of our target species. Therefore, our projections should be interpreted with caution. Second, we did not consider the effects of food and habitat competition among specialist insects on their range dynamics, which may, to a certain extent, overestimate their potential ranges. Third, we did not take into account the effects of the specialist insects on the potential ranges of their host tree, which may also overestimate the host’s potential ranges. Additionally, some differences in detectability among our specialist insects, which might induce sample bias among them, could result in differences in the number of occurrences. This might preclude comparison of the potential ranges of the specialist insects, although we showed that differences in the number of occurrence records did not substantially alter the comparability of their future range dynamics.

## 5. Conclusions

We examined shifts in the potential ranges of black locust and its specialist insects in Europe under current–future scenarios and the factors driving these range shifts. We detected range expansions in all target species. Climate predictors and host plant availability were expected to have the strongest effects on the range shifts of the host and its specialist insects, respectively. The main areas of range expansion for all five species were identified in Finland, the United Kingdom, and Poland, suggesting that these areas require special attention to minimize the effects of these invasive species on ecosystems. Reducing climate change in the future is essential for combatting invasions of these specialist insects in Europe given that the range shifts of specialist insects were consistent with those of their host plants; climate change in the future is expected to be the major factor driving host range expansion.

## Data Availability

We have provided all data in the Supplementary Materials.

## Supplementary Materials

The following supporting information can be downloaded at: https://www.mdpi.com/article/10.3390/insects15100765/s1, S1 Spatially thinned occurrences of black locust and its specialist insects in Europe. S2 Variable importance scores in the preliminary models. S3 Multi-collinearity among predictors. S4 Ten algorithms in the models. S5 Retained algorithms in the formal models. S6 Model reliability. S7 Variable importance scores in the formal models. S8 Thresholds for calibrating the ranges.

## References

[1] Vítková M., Müllerová J., Sádlo J., Pergl J., Pyšek P. (2017). Black locust (*Robinia pseudoacacia*) beloved and despised: A story of an invasive tree in Central Europe. For. Ecol. Manag..

[2] Nicolescu V.-N., Hernea C., Bakti B., Keserű Z., Antal B., Rédei K. (2018). Black locust (*Robinia pseudoacacia* L.) as a multi-purpose tree species in Hungary and Romania: A review. J. For. Res..

[3] Nicolescu V.N., Rédei K., Mason W.L., Vor T., Pöetzelsberger E., Bastien J.C., Brus R., Bencat T., Dodan M., Cvjetkovic B. (2020). Ecology, growth and management of black locust (*Robinia pseudoacacia* L.), a non-native species integrated into European forests. J. For. Res..

[4] Li G., Xu G., Guo K., Du S. (2014). Mapping the global potential geographical distribution of black locust (*Robinia pseudoacacia* L.) using herbarium data and a maximum entropy model. Forests.

[5] Martin G.D. (2019). Addressing geographical bias: A review of *Robinia pseudoacacia* (black locust) in the southern hemisphere. S. Afr. J. Bot..

[6] Benesperi R., Giuliani C., Zanetti S., Gennai M., Lippi M.M., Guidi T., Nascimbene J., Foggi B. (2012). Forest plant diversity is threatened by *Robinia pseudoacacia* (black-locust) invasion. Biodivers. Conserv..

[7] Rumlerová Z., Vilà M., Pergl J., Nentwig W., Pyšek P. (2016). Scoring environmental and socioeconomic impacts of alien plants invasive in Europe. Biol. Invasions.

[8] Kroftová M., Reif J. (2017). Management implications of bird responses to variation in non-native/native tree ratios within central European forest stands. For. Ecol. Manag..

[9] Lazzaro L., Mazza G., d’Errico G., Fabiani A., Giuliani C., Inghilesi A.F., Lagomarsino A., Landi S., Lastrucci L., Pastorelli R. (2018). How ecosystems change following invasion by *Robinia pseudoacacia*: Insights from soil chemical properties and soil microbial, nematode, microarthropod and plant communities. Sci. Total Environ..

[10] Nentwig W., Bacher S., Kumschick S., Pyšek P., Vilà M. (2018). More than “100 worst” alien species in Europe. Biol. Invasions.

[11] Nascimbene J., Benesperi R., Casazza G., Chiarucci A., Giordani P. (2020). Range shifts of native and invasive trees exacerbate the impact of climate change on epiphyte distribution: The case of lung lichen and black locust in Italy. Sci. Total Environ..

[12] Mally R., Ward S.F., Trombik J., Buszko J., Medzihorsky V., Liebhold A.M. (2021). Non-native plant drives the spatial dynamics of its herbivores: The case of black locust (*Robinia pseudoacacia*) in Europe. NeoBiota.

[13] Puchałka R., Dyderski M.K., Vítková M., Sádlo J., Klisz M., Netsvetov M., Prokopuk Y., Matisons R., Mionskowski M., Wojda T. (2021). Black locust (*Robinia pseudoacacia* L.) range contraction and expansion in Europe under changing climate. Glob. Chang. Biol..

[14] Tytar V., Nekrasova O., Marushchak O., Pupins M., Skute A., Čeirāns A., Kozynenko I. (2022). The spread of the invasive locust digitate leafminer *Parectopa robiniella* Clemens, 1863 (Lepidoptera: Gracillariidae) in Europe, with special reference to Ukraine. Diversity.

[15] Moshki A., Lamersdorf N.P. (2011). Growth and Nutrient Status of Introduced Black Locust (*Robinia pseudoacacia* L.) Afforestation in arid and semi arid areas of Iran. Res. J. Environ. Sci..

[16] Cierjacks A., Kowarik I., Joshi J., Hempel S., Ristow M., von der Lippe M., Weber E. (2013). Biological flora of the British Isles: *Robinia pseudoacacia*. J. Ecol..

[17] Liu X., Fan Y., Long J., Wei R., Kjelgren R., Gong C., Zhao J. (2013). Effects of soil water and nitrogen availability on photosynthesis and water use efficiency of *Robinia pseudoacacia* seedlings. J. Environ. Sci..

[18] Zhang W., Liu W., Xu M., Deng J., Han X., Yang G., Feng Y.Z., Ren G.X. (2019). Response of forest growth to C: N: P stoichiometry in plants and soils during *Robinia pseudoacacia* afforestation on the Loess Plateau, China. Geoderma.

[19] Rédei K., Csiha I., Keserű Z., Kamandiné Végh Á., Győri J. (2011). The silviculture of black locust (*Robinia pseudoacacia* L.) in Hungary: A review. SEEFOR-South-East Eur. For..

[20] Usta A., Yilmaz M. (2022). Topographic controls in the distributions of tree species on the Karadağ Massif, NE Turkey. Balt. For..

[21] Dyakov N.R. (2016). Spatial distribution of some alien plants across a restricted mountainous area. Contemp. Probl. Ecol..

[22] Vítková M., Tonika J., Müllerová J. (2015). Black locust–successful invader of a wide range of soil conditions. Sci. Total Environ..

[23] Sádlo J., Vítková M., Pergl J., Pyšek P. (2017). Towards site-specific management of invasive alien trees based on the assessment of their impacts: The case of *Robinia pseudoacacia*. NeoBiota.

[24] Richardson D.M., Pyšek P., Rejmánek M., Barbour M.G., Panetta F.D., West C.J. (2000). Naturalization and invasion of alien plants: Concepts and definitions. Divers. Distrib..

[25] Fodor E., Hâruţa O. (2009). Niche partition of two invasive insect species, *Parectopa robiniella* (Lepidoptera; Gracillariidae) and *Phyllonorycter robiniella* (Clem.) (Lepidoptera: Gracillariidae). Res. J. Agric. Sci..

[26] Medzihorský V., Trombik J., Parectopa R., Turčáni M., Liebhold A.M. (2023). Insect invasions track a tree invasion: Global distribution of black locust herbivores. J. Biogeogr..

[27] Staszak A.M., Ratajczak E., Leśniewska J., Piotrowska-Niczyporuk A., Kostro-Ambroziak A. (2023). A broad spectrum of host plant responses to the actions of the gall midge: Case study of *Robinia pseudoacacia* L. and *Obolodiplosis robiniae* (Haldeman). BMC Plant Biol..

[28] Huemer P., Mayr T. (2022). *Chrysaster ostensackenella* (Fitch, 1859), a potentially invasive species newly recorded from Europe (Lepidoptera, Gracillariidae). Check List..

[29] Ivinskis P., Rimšaitė J. (2008). Records of *Phyllonorycter robiniella* (Clemens, 1859) and *Parectopa robiniella* Clemens, 1863 (Lepidoptera, Gracillariidae) in Lithuania. Acta Zool Litu..

[30] Tóth P., Váňová M., Lukáš J. (2011). Impact of natural enemies on *Obolodiplosis robiniae* invasion. Biologia.

[31] Netoiu C., Tomescu R. (2006). Moliile miniere ale salcâmului (*Parectopa robiniella* Clemens, 1863 si Phyllonorycter robiniella Clemens 1859, Lepidoptera, Gracillariidae). Analele ICAS.

[32] Zhao J.Q., Gao T., Du J.J., Shi J. (2023). Future trends in *Obolodiplosis robiniae* distribution across Eurasian continent under global climate change. Insects.

[33] Olenici N., BĂLĂCenoiu F., Tomescu R., NeȚOiu C., Buzatu A., Alexandru A. (2022). Invasive alien forest insect species in south-eastern Romania. Not. Bot. Horti Agrobot..

[34] Chardon N.I., Cornwell W.K., Flint L.E., Flint A., Ackerly D. (2015). Topographic, latitudinal and climatic distribution of *Pinus coulteri*: Geographic range limits are not at the edge of the climate envelope. Ecography.

[35] Li Y., Li X., Sandel B., Blank D., Liu Z.T., Liu X. (2016). Climate and topography explain range sizes of terrestrial vertebrates. Nat. Clim. Chang..

[36] Michalak J.L., Lawler J.J., Roberts D.R., Carroll C. (2018). Distribution and protection of climatic refugia in North America. Conserv. Biol..

[37] Bartlett J.C., Convey P., Pertierra L.R., Hayward S.A. (2020). An insect invasion of Antarctica: The past, present and future distribution of *Eretmoptera murphyi* (Diptera, Chironomidae) on Signy Island. Insect Conserv. Divers..

[38] Oliver T.H., Morecroft M.D. (2014). Interactions between climate change and land-use change on biodiversity: Attribution problems, risks, and opportunities. Wires Clim. Chang..

[39] Bessa A.S., Carvalho J., Gomes A., Santarem F. (2016). Climate and land-use drivers of invasion: Predicting the expansion of *Vespa velutina nigrithorax* into the Iberian Peninsula. Insect Conserv. Divers..

[40] Hill M.P., Gallardo B., Terblanche J.S. (2017). A global assessment of climatic niche shifts and human influence in insect invasions. Glob. Ecol. Biogeogr..

[41] Guo Q., Fei S., Potter K.M., Liebhold A.M., Wen J. (2019). Tree diversity regulates forest pest invasion. Proc. Natl. Acad. Sci. USA.

[42] Zhang X., Nie P., Hu X., Feng J. (2024). Future range expansions of invasive wasps suggest their increasing impacts on global apiculture. Insects.

[43] Fick S.E., Hijmans R.J. (2017). WorldClim 2: New 1-km spatial resolution climate surfaces for global land areas. Int. J. Climatol..

[44] Zhang M.Z., Xu Z., Han Y., Guo W. (2022). Evaluation of CMIP6 models toward dynamical downscaling over 14 CORDEX domains. Clim. Dyn..

[45] Thuiller W., Lafourcade B., Engler R., Araújo M.B. (2009). BIOMOD—A platform for ensemble forecasting of species distributions. Ecography.

[46] Barbet-Massin M., Jiguet F., Albert C.H., Thuiller W. (2012). Selecting pseudo-absences for species distribution models: How, where and how many?. Methods Ecol. Evol..

[47] Nie P., Feng J. (2023). Niche and Range Shifts of *Aedes aegypti* and *Ae. albopictus* Suggest That the Latecomer Shows a Greater Invasiveness. Insects.

[48] Liu C., Newell G., White M. (2016). On the selection of thresholds for predicting species occurrence with presence-only data. Ecol. Evol..

[49] Ciuvăț A.L., Abrudan I.V., Ciuvăț C.G., Marcu C., Lorenț A., Dincă L., Szilard B. (2022). Black locust (*Robinia pseudoacacia* L.) in Romanian forestry. Diversity.

[50] Canelles Q., Bassols E., Vayreda J., Brotons L. (2021). Predicting the potential distribution and forest impact of the invasive species *Cydalima perspectalis* in Europe. Ecol. Evol..

[51] Lach L. (2021). Invasive ant establishment, spread, and management with changing climate. Curr. Opin. Insect Sci..

[52] Wang C.J., Wang S.J., Yu C.M., Wang X.T., Wang R., Wan J.Z. (2023). Habitat heterogeneity and topographic variation as the drivers of insect pest distributions in alpine landscapes. Acta Ecol. Sin..

[53] Wang L., Zhang F.X., Li L.P., Wang C.J., Wan J.Z. (2023). Effects of habitat heterogeneity and topographic variation on insect pest risks in alpine regions. Land.

[54] Baidya P., Bagchi S. (2022). Influence of human land use and invasive species on beta diversity of tropical ant assemblages. Insect Conserv. Divers..

[55] Della Rocca F., Milanesi P. (2022). The new dominator of the world: Modeling the global distribution of the Japanese beetle under land use and climate change scenarios. Land.

[56] Montgomery K., Walden-Schreiner C., Saffer A., Jones C., Seliger B.J., Worm T., Tateosian L., Shukunobe M., Kumar S., Meentemeyer R.K. (2023). Forecasting global spread of invasive pests and pathogens through international trade. Ecosphere.

[57] Zhou Y., Wu C., Nie P., Feng J., Hu X. (2024). invasive pest and invasive host: Where might spotted-wing drosophila (*Drosophila suzukii*) and American black cherry (*Prunus serotina*) cross paths in Europe?. Forests.

[58] Acosta A.N.C., Mooney K. (2021). Effects of geographic variation in host plant resources for a specialist herbivore’s contemporary and future distribution. Ecosphere.

[59] Liu T.M., Wang J.M., Hu X.K., Feng J.M. (2020). Land-use change drives present and future distributions of Fall armyworm, *Spodoptera frugiperda* (JE Smith) (Lepidoptera: Noctuidae). Sci. Total Environ..

[60] Liebhold A.M., Yamanaka T., Roques A., Augustin S., Chown S.L., Brockerhoff E.G., Pyšek P. (2018). Plant diversity drives global patterns of insect invasions. Sci. Rep..

[61] Sirami C., Caplat P., Popy S., Clamens A., Arlettaz R., Jiguet F., Brotons L., Martin J.L. (2017). Impacts of global change on species distributions: Obstacles and solutions to integrate climate and land use. Glob. Ecol. Biogeogr..

[62] Begum S., Nakaba S., Yamagishi Y., Oribe Y., Funada R. (2013). Regulation of cambial activity in relation to environmental conditions: Understanding the role of temperature in wood formation of trees. Physiol. Plant..

[63] Hacket-Pain A.J., Ascoli D., Vacchiano G., Biondi F., Cavin L., Conedera M., Drobyshev I., Liñán I.D., Friend A.D., Grabner M. (2018). Climatically controlled reproduction drives interannual growth variability in a temperate tree species. Ecol. Lett..

[64] De Micco V., Carrer M., Rathgeber C.B.K., Julio Camarero J., Voltas J., Cherubini P., Battipaglia G. (2019). From xylogenesis to tree rings: Wood traits to investigate tree response to environmental changes. IAWA J..

[65] Friend A.D., Eckes-Shephard A.H., Fonti P., Rademacher T.T., Rathgeber C.B.K., Richardson A.D., Turton R.H. (2019). On the need to consider wood formation processes in global vegetation models and a suggested approach. Ann. For. Sci..

