# Supplementary material for: A Host Tree and Its Specialist Insects: Black Locust (Robinia pseudoacacia) Availability Largely Determines the Future Range Dynamics of Its Specialist Insects in Europe
Source: Insects. 2024 Oct 2;15(10):765. doi: 10.3390/insects15100765 (PMC11514610; doi:10.3390/insects15100765)
Supplement: Supplementary file 1 [file insects-15-00765-s001.zip › Supplemental Material S4.pdf]

S4 Ten algorithms in the models.

We adopted the following ten algorithms in our SDMs: Surface Range Envelope, Random Forest, Multiple Adaptive Regression Splines, XGBoost, Maximum Entropy Modeling, Artificial Neural Network, Generalized Linear Model, Classification Tree Analysis, Flexible Discriminant Analysis, and the Generalized Boosting Model
